# Bidirectional coordination of actions and habits by TrkB in mice

**DOI:** 10.1038/s41598-018-22560-x

**Published:** 2018-03-14

**Authors:** Elizabeth G. Pitts, Dan C. Li, Shannon L. Gourley

**Affiliations:** 1 Graduate Program in Neuroscience, Yerkes National Primate Research Center, Emory University, Atlanta, GA USA; 2 Departments of Pediatrics and Psychiatry and Behavioral Sciences, Emory University School of Medicine, Emory University, Atlanta, GA USA

## Abstract

Specific corticostriatal structures and circuits are important for flexibly shifting between goal-oriented versus habitual behaviors. For example, the orbitofrontal cortex and dorsomedial striatum are critical for goal-directed action, while the dorsolateral striatum supports habits. To determine the role of neurotrophin signaling, we overexpressed a truncated, inactive form of tropomyosin receptor kinase B [also called tyrosine receptor kinase B (TrkB)], the high-affinity receptor for Brain-derived Neurotrophic Factor, in the orbitofrontal cortex, dorsomedial striatum and dorsolateral striatum. Overexpression of truncated TrkB interfered with phosphorylation of full-length TrkB and ERK42/44, as expected. In the orbitofrontal cortex and dorsomedial striatum, truncated trkB overexpression also occluded the ability of mice to select actions based on the likelihood that they would be reinforced. Meanwhile, in the dorsolateral striatum, truncated trkB blocked the development of habits. Thus, corticostriatal TrkB-mediated plasticity appears necessary for balancing actions and habits.

## Introduction

Flexible action requires shifting between familiar and novel behavioral strategies. Extensive response training and exposure to stressors and certain drugs of abuse can lead to a bias towards habit-based behaviors that are by contrast inflexible. Maladaptive habits may contribute to illnesses characterized by impulse control deficits, such as addiction and obsessive-compulsive disorder^1,2^. Nevertheless, the mechanisms by which the brain balances actions and habits are still being identified.

During the initial acquisition of an instrumental behavior, organisms are typically sensitive to the predictive relationship between actions and their outcomes, and goal-directed action selection strategies dominate. After continued training, reward-related stimuli can gain control over behavior, and behavioral response strategies become automated, or “habitual,” and insensitive to action-outcome associations^3–5^. The posterior dorsomedial striatum (DMS) and orbitofrontal prefrontal cortex (oPFC) are necessary for goal-directed actions, while the dorsolateral striatum (DLS) controls habits^3,6–8^.

The primary neurotrophin Brain-derived Neurotrophic Factor (BDNF) appears to be a key cortical substrate coordinating goal-directed action selection, given that oPFC-selective *Bdnf* knockdown causes failures in action-outcome decision making and a deferral to habit-based behaviors^7,9^. Where, precisely, stimulation of the high-affinity BDNF receptor tyrosine receptor kinase B (TrkB) is important remains unclear, however, given that BDNF is subject to anterograde transport. For example, oPFC-selective *Bdnf* knockdown reduces BDNF protein in the dorsal striatum^7^, suggesting that TrkB activation in the oPFC, DMS, or both could support goal-directed action. Resolving these possibilities is important because upon BDNF binding, the intracellular domain of TrkB auto-phosphorylates, creating docking sites for effector proteins that initiate intracellular signaling cascades, *e.g*., the ERK42/44 and Akt pathways. TrkB impacts a diverse array of neuronal functions including cell survival and differentiation, axonal and dendritic growth and arborization and synapse formation and plasticity.

**Figure 1:**
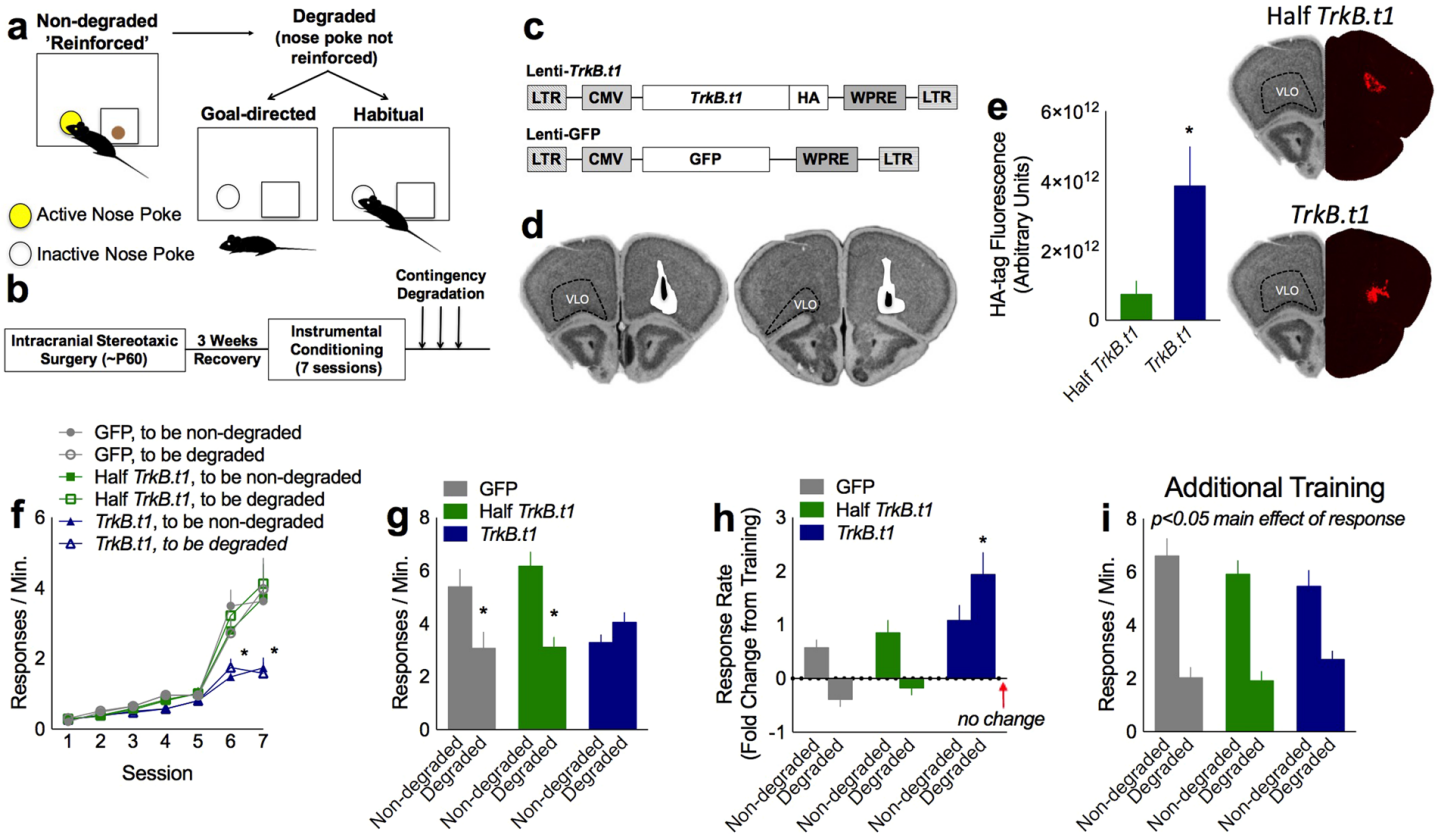
*TrkB.t1* overexpression in the oPFC impedes goal-directed action selection. (**a**) Behavioral testing approach: Mice were trained to nose poke on two ports for food reinforcers. Then, one response was reinforced approximately 50% of the time (‘Non-degraded’), while the probability of reinforcement associated with the other response was greatly decreased (‘Degraded’), given that pellets were delivered non-contingently. Inhibiting responding in this condition is considered goal-directed, while insensitivity to non-contingent pellet delivery is considered habitual. (**b**) Experimental timeline: Mice were infused with viral vectors, then behaviorally tested. (**c**) Viral vector constructs (from ref.^10^) are shown. (**d**) A lentivirus expressing *TrkB.t1*, GFP, or a half-and-half mixture of both was infused bilaterally into the oPFC. Representative viral vector spread is represented on images from the Mouse Brain Library^27^. White represents the maximal spread and black the smallest. “VLO” refers to the ventrolateral oPFC. (**e**) Quantitative immunostaining revealed that full-titer lenti-*TrkB.t1* infusions generated greater HA immunofluorescence than a half-and-half mixture of lenti-GFP and lenti-*TrkB.t1* (“Half-*TrkB.t1*”) (GFP: n = 4; *TrkB.t1*: n = 5). **Inset:** Representative HA immunofluorescence. (**f**) Mice were trained to respond for food reinforcers. Full-titer *TrkB.t1* overexpression reduced response rates (n = 6 mice/group). (**g**) Further, mice with full-titer lenti-*TrkB.t1* were insensitive to action-outcome contingencies, failing to reduce responding when responding was not reinforced. (**h**) The same data were normalized to response rates generated on the last day of training, such that 0 reflects no change. Response rates increased in the ‘Non-degraded’ condition across groups. Meanwhile, full-titer *TrkB.t1* overexpression interfered with response inhibition, such that these mice maintained high levels of responding even when responding was not reinforced (‘Degraded’ condition). (**i**) With additional exposure to noncontingent pellet delivery, full-titer *TrkB.t1* mice were ultimately able to inhibit a nonreinforced response. Bars and symbols represent means + SEMs, *p < 0.05. Behavioral findings are concordant with independent unpublished pilot investigations and post-mortem experiments were conducted at least twice.

Here, mice were trained to generate two food-reinforced behaviors in operant conditioning chambers, then tested for sensitivity to action-outcome associations using a contingency degradation procedure. In this task, food pellets associated with one familiar behavior are delivered non-contingently (“for free”), regardless of the animal’s actions, while the other response remains reinforced (Fig. 1a). Mice that are sensitive to action-outcome contingencies decrease responding during the ‘degraded’ session since responding is not rewarded. By contrast, nose poking that has taken on habitual qualities remains robust.

First, we infused into the oPFC a lentivirus expressing a truncated, inactive form of TrkB, *TrkB.t1*, which lacks an intracellular domain and therefore cannot initiate intracellular signaling pathways (from^10^). We infused lenti-*TrkB.t1* with an HA tag, lenti-Green Fluorescent Protein (GFP; a control), or a half-and-half mixture of the two in order to generate multiple lenti-*TrkB.t1* “doses” (Fig. 1b–d). The full-titer lenti-*TrkB.t1* infusion generated significantly greater HA immunoreactivity than the low-titer mixture (“Half *TrkB*.t1”) (*t*_7_ = −2.357, p = 0.05) (Fig. 1e), as expected. We then trained mice to nose poke for food reinforcers. While all groups initially acquired the responses, mice with full-titer lenti-*TrkB.t1* generated lower response rates (day * group interaction *F*_12,90_ = 5.565, *p* < 0.001; main effects: day *F*_6,90_ = 86.345, *p* < 0.001; nose poke *F*_1,15_ = 0.284, *p* = 0.602; group *F*_2,15_ = 5.816, *p* = 0.013) (Fig. 1f), as also occurs with oPFC-selective *Bdnf* knockdown^7,9^ and oPFC damage more generally^11^. This profile is also consistent with impaired action-outcome decision making^12^. Indeed, lenti-*TrkB.t1* interfered with the ability of mice to select actions based on their consequences during an instrumental contingency degradation procedure (Fig. 1g). Specifically, lenti-*TrkB.t1* mice failed to inhibit a response that was unlikely to be rewarded. In contrast, GFP control and low-titer mice decreased responding when that behavior was unlikely to be reinforced (interaction *F*_2,15_ = 6.191, *p* = 0.011; main effects: nose poke *F*_1,15_ = 10.816, *p* = 0.005; group *F*_2,15_ = 2.042, *p* = 0.164) (Fig. 1g).

Given low response rates during instrumental response training (Fig. 1f), it is conceivable that full-titer lenti-*TrkB.t1* mice were simply unable to energize a response that was reinforced (*i.e*., as opposed to being unable to inhibit an inappropriate response). We feel that this is unlikely, however, given that across all groups, response rates during the reinforced phase of testing were *higher* than those generated on the last day of training (main effect of test phase *F*_1,15_ = 14.696, *p* = 0.002; no interactions) (Fig. 1h). Groups differed only during the “degradation” phase when responding was no longer reinforced (Fig. 1h). Again, full-titer lenti-*TrkB.t1* mice generated response rates higher than the last day of training (1-sample t-test against no change (0) *t*_5_ = 4.385, p = 0.007), even though responding was not reinforced. By contrast, the other groups did not (GFP control 1-sample t-test against no change (0) *t*_4_ = −2.414, p = 0.073; Half *TrkB.t1* 1-sample t-test against no change (0) *t*_5_ = −1.233, p = 0.272).

Does *TrkB.t1* overexpression in the oPFC block, or instead delay, action-outcome learning and memory? To answer this question, we exposed mice to non-contingent pellet delivery for 2 additional sessions. Ultimately (in the final session), full-titer *TrkB.t1* mice inhibited nonreinforced responding in a goal-directed manner, indicating that *TrkB.t1* overexpression delayed, but did not fully occlude, action-outcome-based decision making (main effects: nose poke *F*_1,15_ = 61.688, *p* < 0.001; group *F*_2,15_ = 0.418, *p* = 0.666; no interactions) (Fig. 1i).

**Figure 2:**
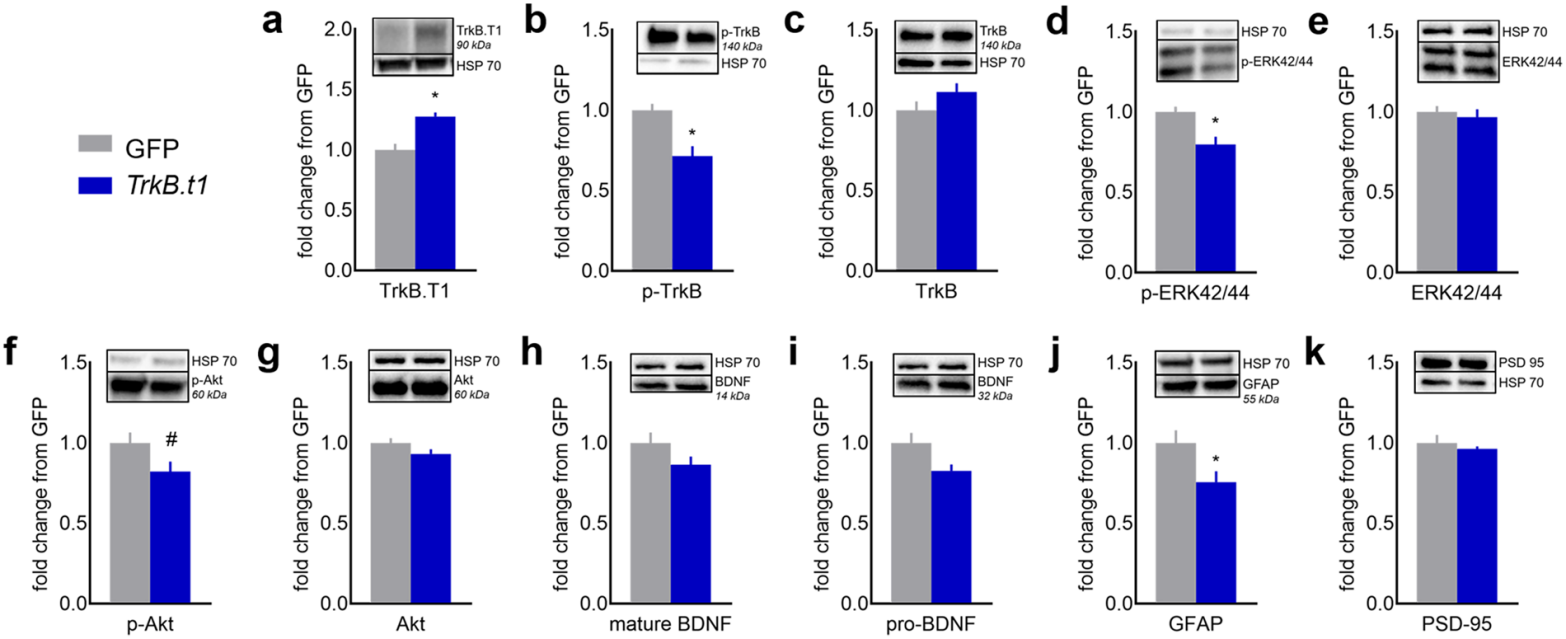
Validation of the *TrkB.t1*-overexpressing virus. (**a**) Virus-infected oPFC tissue was dissected by tissue punch and immunoblotted for TrkB.T1, revealing elevated TrkB.T1 protein in mice bearing the *TrkB.t1*-overexpressing virus, as expected (GFP: n = 4; *TrkB.t1*: n = 5). (**b**) Phospho-TrkB was also diminished (GFP: n = 12; *TrkB.t1:* n = 16, representing 2 independent cohorts; applies also to all following panels). (**c**) Full-length TrkB was unaffected. (**d**) Phospho-ERK42/44 was reduced, while (**e**) total ERK42/44 was unchanged. (**f**) Similarly, we identified a trend for reduced phospho-Akt and (**g**) no changes in total Akt. (h) Mature BDNF and (**i**) the pro-form were not significantly affected. (**j**) Finally, the astrocytic marker GFAP was reduced and (**k**) the synaptic marker PSD95 was not affected. Representative, unadjusted lanes from the same individual gels are shown with their corresponding loading controls. Molecular weights of each protein are indicated either in, or directly adjacent to, the protein name. Bars represent means + SEMs, *p < 0.05, ^#^p = 0.06. Every gel was run at least twice.

We next generated additional mice with either lenti-GFP or full-titer lenti-*TrkB.t1* in the oPFC. We euthanized them 3 weeks following viral vector infusion and extracted the oPFC using a tissue punch. In this case, tissue samples would be expected to contain both infected and uninfected cells. Nevertheless, Trkb.T1 protein levels were detectably elevated in mice bearing the lenti-*TrkB.t1* virus, as would be expected (*t*_7_ = −2.769, p = 0.028) (Fig. 2a). Phospho-TrkB was also diminished, consistent with the notion that over-expression of a truncated receptor interferes with signaling of the full-length receptor^13–15^ (t_26_ = 3.575, p = 0.001) (Fig. 2b). Meanwhile, total full-length TrkB protein was unaffected, indicating no gross compensatory changes in receptor expression (t_26_ = −1.453, p = 0.158) (Fig. 2c). Consistent with reductions in phospho-TrkB, phospho-ERK42/44 was also diminished (t_26_ = 3.218, p = 0.003) (Fig. 2d; agrees with ref.^16^, which uses the same viral vector in the hippocampus), while total ERK42/44 was unchanged (t_26_ = 0.493, p = 0.626) (Fig. 2e). Similarly, we identified a trend for reduced phospho-Akt (t_26_ = 3.218, p = 0.061) (Fig. 2f) and no changes in total Akt (t_26_ = 1.547, p = 0.134) (Fig. 2g).

BDNF levels can dynamically impact reward-related decision making. For example, microRNA regulation of BDNF in the prefrontal cortex mediates escalating alcohol intake in mice^17^. To address the possibility that *TrkB.t1* overexpression led to an accumulation of cortical BDNF (*e.g*., by interfering with axonal transport), or alternatively, diminished local BDNF levels, we also quantified BDNF. Neither mature nor pro-BDNF were significantly affected, despite large group sizes (though downward trends were noted: t_26_ = 1.651, p = 0.111; t_26_ = 2.135, p = 0.090, respectively) (Fig. 2h and i). To address the potential concern that *TrkB.t1* overexpression caused lesion-like tissue damage, we quantified the astrocytic marker GFAP, which increases upon lesion. *TrkB.t1* overexpression reduced GFAP levels, however (t_26_ = 2.411, p = 0.027) (Fig. 2j). Our final finding that the postsynaptic marker PSD95 was not affected (t_26_ = 0.757, p = 0.457) (Fig. 2k) further supports our perspective that *TrkB.t1* overexpression did not cause gross tissue damage.

### As with cortical TrkB, striatal TrkB influences action selection strategies

**Figure 3:**
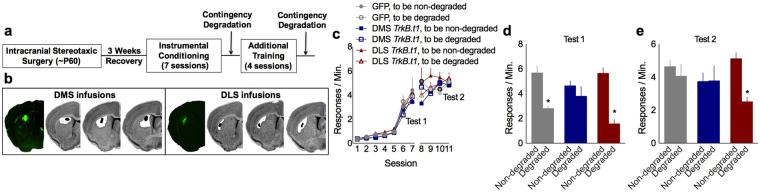
*TrkB.t1* overexpression in the striatum bidirectionally regulates actions and habits. (**a**) Lenti-*TrkB.t1* or GFP was infused into the DMS or DLS, then sensitivity to action-outcome contingency was tested. (**b**) Viral vector infusions are represented, with white representing the largest infusion and black the smallest. (**c**) We detected no group differences during food-reinforced instrumental conditioning. (**d**) Overexpression of *TrkB.t1* in the DMS, however, caused a bias towards inflexible habits, indicated by insensitivity to action-outcome contingencies. (**e**) Additional nose poke training induced habits in control mice, but overexpression of *TrkB.t1* in the DLS blocked these habits from forming. n = 8, 6, 7 for GFP, DMS and DLS, respectively. Bars and symbols represent means + SEMs, *p < 0.05. Results are concordant with independent unpublished pilot investigations.

Obstructing oPFC-striatal interactions causes the same impairments in goal-directed action as with oPFC-selective *TrkB.t1* overexpression here^7^. Interfering with oPFC-striatal interactions also impedes an organism’s ability to modify instrumental behaviors when reward value changes^8^. The striatum contains very little *Bdnf* mRNA^18^, but abundant BDNF protein anterogradely transported from cortical sources^19^. We thus next examined whether TrkB in the dorsal striatum is similarly important for flexible action selection. In this case, we overexpressed *TrkB.t1* selectively in the DMS or DLS (using the full-titer viral vector also used in Figs 1 and 2) (Fig. 3a,b). Response rates during initial nose poke training did not differ between groups (main effects: day *F*_10,180_ = 112.669, *p* < 0.001; nose poke *F*_1,18_ = 0.006, *p* = 0.937; group *F*_2,18_ = 0.674, *p* = 0.522) (Fig. 3c). *TrkB.t1* overexpression in the DMS, however, induced failures in goal-oriented response selection, causing robust response rates despite non-contingent delivery of food pellets (interaction *F*_2,18_ = 8.14, *p* = 0.003; main effects: nose poke *F*_1,18_ = 65.625, *p* < 0.001; group *F*_2,18_ = 0.918, *p* = 0.417) (Fig. 3d). Thus, TrkB in the oPFC and downstream DMS appears to be essential for goal-directed action.

Next, we *induced* habit behavior using a random interval schedule of reinforcement (Fig. 3a). Following this training, both control and DMS *TrkB.t1* mice generated inflexible habit-based responding as expected, indicated by insensitivity to action-outcome contingencies. By contrast, *TrkB.t1* overexpression in the DLS interfered with habit formation – these mice remained sensitive to changes in action-outcome contingencies despite extensive behavioral experience (interaction *F*_2,17_ = 4.198, *p* = 0.033; main effects: nose poke *F*_1,17_ = 7.693, *p* = 0.013; group *F*_2,17_ = 0.495, *p* = 0.618) (Fig. 3e).

To summarize, TrkB appears to be essential to the functions of both the DMS (supporting goal-directed action) and DLS (supporting habits). Indeed, *TrkB.t1* overexpression in these striatal sub-regions causes response patterns that bear remarkable resemblance to those following inactivation of each respective structure^20,21^. Although TrkB is expressed in both the DMS and DLS^22^, these patterns were nevertheless somewhat unexpected, given that systemic administration of a putative TrkB agonist blocks habits induced by extensive response training^9^ and excess glucocorticoids^16^, rather than *facilitating* this DLS-dependent behavior. TrkB stabilizes dendritic spine densities and morphologies throughout multiple brain regions^23^ and is essential for corticostriatal long-term potentiation^24^. The switch from goal-directed action to habits is thought to reflect a transition in the coordinated control of response strategies by multiple cortico-striatal regions to a predominantly DLS-controlled output (*e*.*g*.,^3^). Thus, broad-spread TrkB stimulation (*i.e*., due to systemic injection of a TrkB agonist) may energize goal-directed action by stimulating multiple cortico-striatal structures (such as the oPFC, DMS and prelimbic prefrontal cortex)^4,12^ competing with the DLS for control over behavior. Further understanding the molecular mechanisms mediating the balance between actions and habits could shed light onto treating disorders characterized by impairments in flexible action and decision making, such as obsessive-compulsive disorder and addiction^1,2^.

## Methods

### Subjects

Experiments used adult male wild-type C57BL/6 mice (≥postnatal day 60) (Jackson Laboratories, Bar Harbor, ME). Mice were housed 2–5 per cage and maintained on a 12-hour light cycle (on at 0800) and were experimentally naïve. Mice had *ad libitum* access to water and food, except during instrumental conditioning when body weights were maintained at ~90% of baseline. Procedures were approved by the Emory University Institutional Animal Care and Use Committee and were performed in concordance with *The Guide for the Care and Use of Laboratory Animals*.

### Intracranial surgery

Mice were anaesthetized with ketamine/dexdomitor and then mounted onto a digital stereotaxic apparatus (Stoelting, Wood Dale, IL). Lentiviral vectors expressing *TrkB.t1* and an HA tag or GFP under a CMV promotor were generated by the Emory University Viral Vector Core and have been described in detail previously^10^. Viral vectors were infused at a rate of 0.1 μL/minute, with a total volume of 0.5 μL and the microsyringe left in place for 5 minutes following infusion. In experiments targeting the oPFC, viral vectors were infused at +2.6 mm anteroposterior (AP), −2.85 mm dorsoventral (DV) and +/−1.2 mm mediolateral (ML). Viral vectors targeting the DMS were delivered to +0.74 mm AP, −3.0 mm DV and +/−2.2 mm ML. DLS coordinates were +0.5 mm AP, −3.5 mm DV and +/−2.7 mm ML.

### Action-outcome contingency degradation

Mice were trained to nose poke for food pellet reinforcers (20 mg grain-based pellets; Bioserv, Frenchtown, NJ) in Med-Associates (Georgia, VT) operant conditioning chambers. Mice were trained to nose poke on 2 available apertures using a fixed ratio 1 (FR1) schedule of reinforcement for 5 sessions. Next, mice were trained for 2 additional days using a random interval 30 second (RI30) schedule of reinforcement. Sessions lasted for 70 minutes or until the maximum 60 pellets (30 per nose poke) had been delivered.

Next, mice were tested for sensitivity to action-outcome contingencies using a modified version of classical action-outcome contingency degradation, the details of which are further discussed in refs^25,26^. Briefly, during the ‘non-degraded’ session, one nose poke aperture was occluded and responding on the other nose poke aperture was reinforced using a variable ratio 2 (VR2) schedule of reinforcement. The next day, during the ‘degraded’ session, pellets were delivered non-contingently at a rate yoked to the reinforcement rate from the previous session. Responses were recorded, but had no programmed consequences. The location of the ‘degraded’ aperture was counterbalanced across subjects. Mice that decrease their response rates during the ‘degraded’ session are considered goal-directed. Equivalent response rates during the ‘non-degraded’ and ‘degraded’ sessions are thought to reflect habitual responding^4^.

In experiments with oPFC infusions, mice were tested in the modified contingency degradation procedure 3 consecutive times. In experiments with striatal infusions, following the first contingency degradation test, mice were trained for an additional 4 days with 2 available nose poke recesses using an RI60-second schedule of reinforcement. Then, mice were again tested for sensitivity to action-outcome contingency degradation, as above.

### Immunohistochemistry

#### Histology

Mice were anesthetized by isoflurane and euthanized by rapid decapitation. Brains were stored for 48 hours in 4% paraformaldehyde and then transferred to a 30% w/v sucrose solution. Brains were then sectioned at 50 μM. To verify infusion sites, sections were immunostained for the HA tag on the *TrkB.t1* virus, or GFP was imaged. To visualize HA, sections were blocked, then incubated with the primary antibody [anti-HA; 1:1000; Sigma-Aldrich (Product #H6908), St. Louis, MO] overnight at 4 °C. The next day, sections were incubated with secondary antibody (Alexa Fluor 488 or 594 anti-rabbit; 1:500; Jackson ImmunoResearch Laboratories, West Grove, PA) and then mounted with Permount (Fisher Scientific, Hampton, NH) for fluorescence imaging. Mice with mislocalized infusions were excluded from analysis, resulting in the omission of 1 mouse from each of the *TrkB.t1* groups and 2 mice from the GFP control groups in the oPFC infusion experiment and 2 mice from each group in the dorsal striatal infusion experiment.

#### Quantitative imaging

Sections were immunostained for the HA tag (as above). Sections were imaged on a Nikon 4550 s SMZ18 stereo microscope (Nikon Instruments, Melville, NY). All images were collected in the same session with settings held constant. A sampling area was drawn around the infusion site and the mean integrated intensity was quantified in NIS Elements (Nikon Instruments).

### Western blotting

Behaviorally-naïve mice received oPFC-targeted infusions of full-titer lenti-*TrkB.t1* or GFP as above. Approximately 3 weeks following infusion, matching the onset of behavioral studies, mice were rapidly decapitated and brains were stored at −80 °C, then later sectioned into 1-mm thick sections. The oPFC was dissected using a 1 mm tissue core. Tissue was homogenized in lysis buffer [200 μL; 137 mM NaCl, 20 mM tris-HCl (pH = 8), 1% igepal, 10% glycerol, 1:100 Phosphatase Inhibitor Cocktails 1 and 2 (Sigma-Aldrich) and 1:1000 Protease Inhibitor Cocktail (Sigma-Aldrich)] and protein concentrations were determined by a Pierce BCA Protein Assay kit (Thermo Fisher Scientific). 15 μg of each sample was separated by SDS-page on a 7.5% gradient Tris-glycine gel (Bio-Rad Laboratories, Inc., Hercules, CA). Next, samples were transferred to a PVDF membrane (Bio-Rad) and blocked with 5% nonfat dry milk for 1 hour. The membrane was incubated overnight at 4 °C in primary antibodies. Primary antibodies were TrkB [1:375; Cell Signaling Technology (Product #4606), Danvers, MA], phospho-Trk (Y706/Y707) [1:100; Cell Signaling (Product #4621)], Akt [1:500; Cell Signaling (Product #9271)], phospho-Akt (T308) [1:100; Cell Signaling (Product #2965)], ERK42/44 [1:500; Cell Signaling (Product #9102)], phospho-ERK42/44 (T202/Y204) [1:250; Cell Signaling (Product #4370)], BDNF [1:250; Sigma-Aldrich (Product #B9436)], GFAP [1:1000; Invitrogen (Product #180063)], PSD-95 [1:5000; Cell Signaling (Product #3450)] and HSP70 [1:5000 to 1:10000; Santa Cruz Biotechnology (Product #sc-7298), Dallas, TX]. Following 1 hour of incubation in secondary antibodies [goat anti-mouse and anti-rabbit peroxidase labeled IgG (Vector Laboratories, Burlingame, CA)], immunoreactivity was assessed using a chemiluminescence substrate (Thermo Fisher Scientific) and a ChemiDoc MP Imaging System (Bio-Rad). Immunoblot comparisons were generated at least twice.

### Statistical analyses

All mice were randomly assigned to condition, and sample sizes were in line with prior reports using the same approaches (*e.g*., refs^7,9^). Behavioral response rates were compared by 2-factor mixed-design ANOVA and Bonferroni post-hoc comparisons in case of significant interactions. In an additional analysis, response rates during the instrumental contingency degradation testing phases were normalized to response rates associated with the same nose poke port generated on the final day of training. Fold-change values were compared by 2-factor ANOVA, as well as 1-sample t-tests against no change (0).

For western blotting experiments, densitometry values were normalized to a loading control (HSP70) in the same lane and then to the control sample mean on the same gel to accommodate fluorescence variance *across* gels. Group means were then compared by a 2-tailed unpaired *t-*test.

Throughout, normality was confirmed using the Shapiro-Wilk test. Values >2 standard deviations above or below the mean were considered outliers and excluded, resulting in the omission of 1 mouse each from the fold-change calculations in Fig. 1h and the instrumental contingency degradation test 2 in Fig. 3. Statistical analyses were performed in SPSS or Prism with α ≤ 0.05. Data are presented as mean ± SEM and sample sizes are included in the associated figure legends.

Behavioral experiments were not performed blind to the condition, but response rates were collected via automated photobeam-based systems, minimizing bias. Similarly, equivalent amounts of protein were loaded in western blotting experiments, also minimizing bias.

### Data availability statement

Data can be made available upon reasonable request.
